# Supplementary material for: Combined Effect of Midazolam and Bone Morphogenetic Protein-2 for Differentiation Induction from C2C12 Myoblast Cells to Osteoblasts
Source: Pharmaceutics. 2020 Mar 2;12(3):218. doi: 10.3390/pharmaceutics12030218 (PMC7150865; doi:10.3390/pharmaceutics12030218)
Supplement: Supplementary file 1 [file pharmaceutics-12-00218-s001.pdf]

Supplementary Materials: Combined Effect of Midazolam and Bone Morphogenetic Protein-2 for Differentiation Induction from C2C12 Myoblast Cells to Osteoblasts

Yukihiko Hidaka, Risako Chiba-Okuma, Takeo Karakida, Kazuo Onuma, Ryuji Yamamoto, Keiko Fujii-Abe, Mari M. Saito, Yasuo Yamakoshi and Hiroshi Kawahara

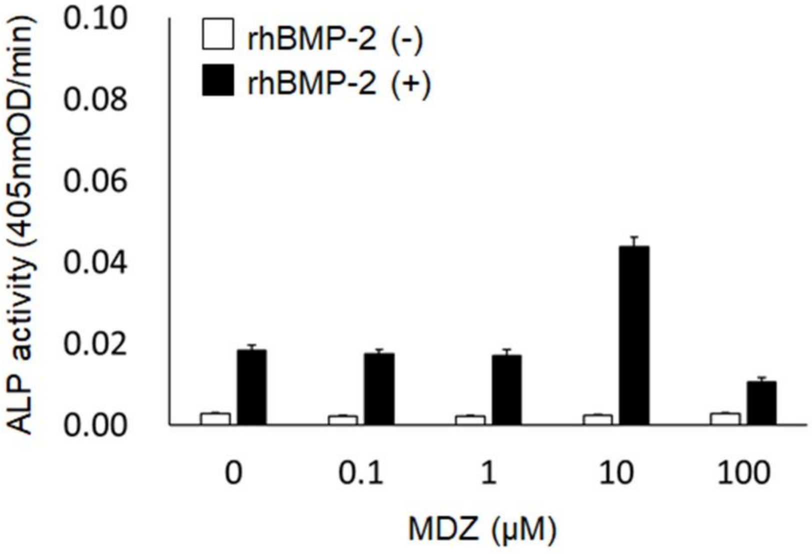

Figure S1. Combined effect of MDZ and BMP-2 on ALP activity in the C2C12 cells. ALP-inducing activity of MDZ (0, 0.1, 1, 10 and 100 μM) with 500 ng/mL rhBMP-2.

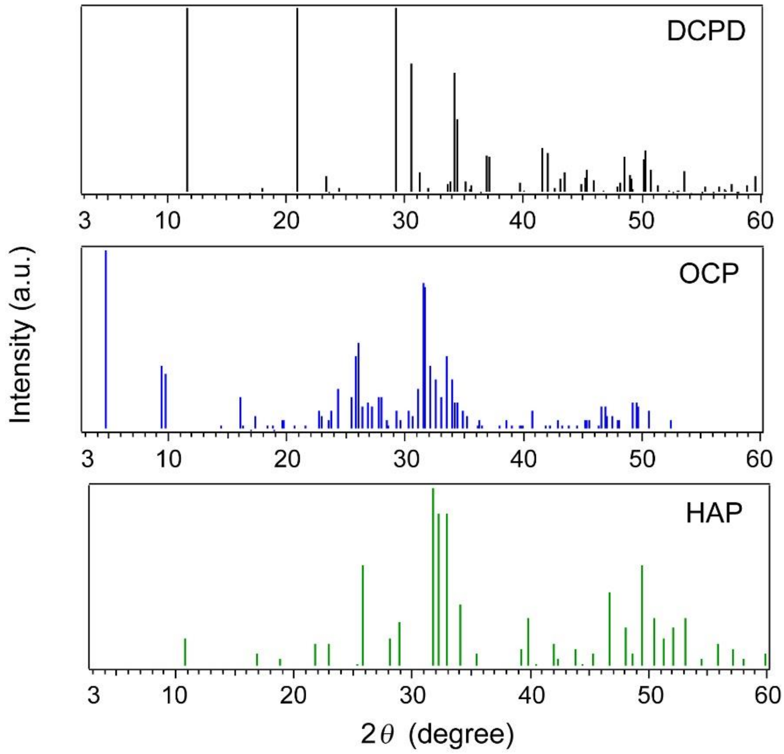

Figure S2. Ideal XRD patterns for DCPD, OCP, and HAP.

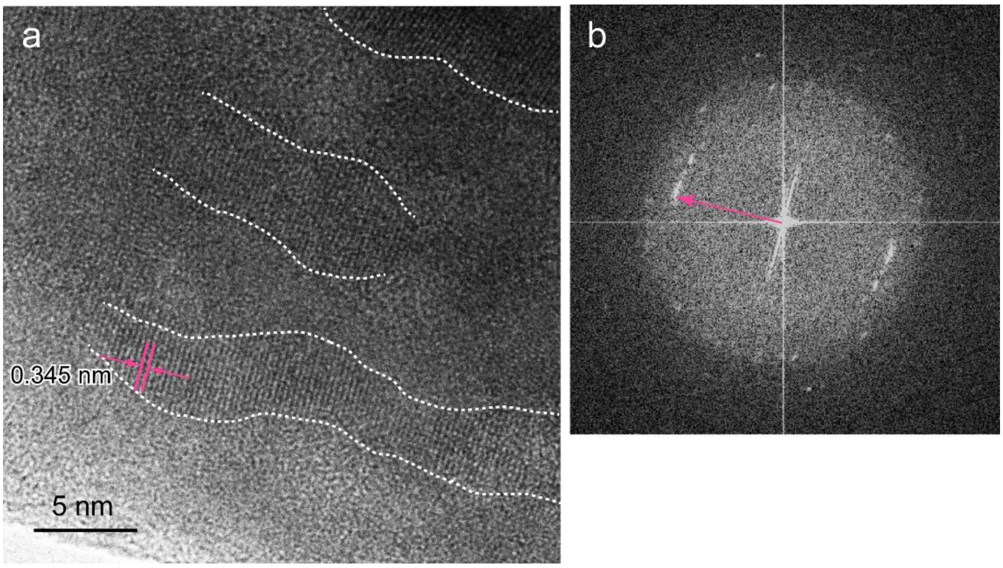

Figure S3. HR-TEM and FFT images of nano-fibers. **(a)** HR-TEM image of nano-fibers. **(b)** FFT image of (a). Magenta arrow direction corresponds to HAP [002]. The *d* of HAP (002) was superimposed in (a).

Table S1. Selected primers, size of amplified product for qPCR analysis shown in main Figure 4.

Gene		Sequence (5'->3')	Size (bp)	qPCR Protocol (45 cycles)
Runx2	F	CCAGATGACATCCCCATCCATC	150	Denaturation 95 °C, 10 sec<br>Annealing 60 °C, 10 sec<br>Extension 72 °C, 15 sec
	R	TTACTGAGAGAGGAAGGCCAGA		
Tnsalp	F	GGGCAATGAGGTCACATCCA	85	
	R	GTGGTTCACCCGAGTGGTAG		
Gapdh	F	CCATCACCATCTTCCAGGAG	346	
	R	ACAGTCTTCTGGGTGGCAGT		
Osx	F	TGGCGTCCTCTCTGCTTGAGG	98	Denaturation 95 °C, 10 sec<br>Annealing 62 °C, 10 sec<br>Extension 72 °C, 15 sec
	R	CAGAGGGCTAGAGCCGCCAA		
MyoD	F	TACAGTGGCGACTCAGATGC	116	
	R	TAGTAGGCGGTGTCGTAGCC		

F: forward, and R: reverse.
